# In-vivo functional and structural retinal imaging using multiwavelength photoacoustic remote sensing microscopy

**DOI:** 10.1038/s41598-022-08508-2

**Published:** 2022-03-16

**Authors:** Zohreh Hosseinaee, Nicholas Pellegrino, Nima Abbasi, Tara Amiri, James A. Tummon Simmons, Paul Fieguth, Parsin Haji Reza

**Affiliations:** 1 PhotoMedicine Labs, Department of System Design Engineering, University of Waterloo, 200 University Ave W, Waterloo, ON N2L 3G1 Canada; 2 Department of System Design Engineering, University of Waterloo, 200 University Ave W, Waterloo, ON N2L 3G1 Canada

**Keywords:** Medical research, Engineering, Optics and photonics

## Abstract

Many important eye diseases as well as systemic disorders manifest themselves in the retina. Retinal imaging technologies are rapidly growing and can provide ever-increasing amounts of information about the structure, function, and molecular composition of retinal tissue in-vivo. Photoacoustic remote sensing (PARS) is a novel imaging modality based on all-optical detection of photoacoustic signals, which makes it suitable for a wide range of medical applications. In this study, PARS is applied for in-vivo imaging of the retina and estimating oxygen saturation in the retinal vasculature. To our knowledge, this is the first time that a non-contact photoacoustic imaging technique is applied for in-vivo imaging of the retina. Here, optical coherence tomography is also used as a well-established retinal imaging technique to navigate the PARS imaging beams and demonstrate the capabilities of the optical imaging setup. The system is applied for in-vivo imaging of both microanatomy and the microvasculature of the retina*.* The developed system has the potential to advance the understanding of the ocular environment and to help in monitoring of ophthalmic diseases.

## Introduction

Functional imaging techniques enable measuring alterations in biological activities, including metabolism, blood flow, regional chemical composition, and biochemical processes. These methods promise to improve the ability to study in-situ biochemistry and disease pathology^1^. In ophthalmology, functional changes most often precede structural changes in major eye diseases. Detecting these alterations aids in understanding pathogenesis, early diagnosis, and timely management of ophthalmic disorders^2^. Retinal oxygen saturation (SO_2_) and metabolic rate of oxygen consumption (MRO_2_) are among the most important biomarkers characterizing the pathophysiological status of the posterior eye. Additionally, abnormal retinal SO_2_ levels are believed to be involved in major eye diseases such as diabetic retinopathy (DR) and age-related macular degeneration (AMD)^2–4^. Therefore, the precise measurement of retinal oxygen saturation can be critical in investigating these blinding diseases. In addition, since the retinal arterioles are derived from the central nervous system, presuming the retinal arterial oxygen content is identical to the systemic circulation, retinal oximetry may provide relevant information on oxygen delivery to the central nervous system. Therefore, determining oxygen saturation in the retinal vessels could enhance the monitoring and treatment of critically ill patients in intensive care^5^. Oxygen-sensitive electrodes and magnetic resonance imaging have been used to measure retinal SO_2_, however these methods are usually restricted to terminal experiments and/or limited by low spatial resolution^3,6^. Phosphorescence lifetime imaging has been also applied to map ocular oxygenation in animal models. Unfortunately, the need to inject fluorescent probes into systematic circulation makes the method inappropriate for human practice^7^.

Recently, researchers have focused on optical imaging-based methods to evaluate retinal oxygenation. Optical imaging methods are capable of non-invasive SO_2_ measurement and they can visualize the spatial distribution of oxygenation with high resolution in ocular environment^8^. Optical measurement of SO_2_ is possible because the two forms of hemoglobin, oxy- and deoxyhemoglobin (HbO_2_ and Hb), have distinct optical absorption properties. Owing to the differences in the absorption spectra of oxy- and deoxyhemoglobin, multi-wavelength imaging methods can assess the SO_2_ in retinal vessels^9^. Currently available optical imaging modalities, such as fundus photography^10^, scanning laser ophthalmoscope (SLO)^11^, and optical coherence tomography (OCT)^12–14^ are scattering based techniques that rely on the back-scattered light from the tissue to form an image. Despite the great progress in these optical imaging techniques, however, they use indirect methods to measure optical absorption. Therefore, the accuracy of these methods is affected by factors such as variation in vessel size^15^, pigmentation^16^, multiple light paths^17^, and vessel wall thickness^9^. For example, in larger vessels the amount of detected backscattered light is much greater than in small vessels^17^, hence the calculation of the optical density and SO_2_ values can be affected in clinical trials^18,19^. Additionally, it has been shown that SO_2_ variations induce changes in vessel diameter^9^, which may further alter the amount of backscattered light from the vessel and, consequently, the SO_2_ measurements.

Photoacoustic microscopy (PAM) has the unique ability to map the direct optical absorption properties with high resolution in biological tissues^20^. The modality has the potential to overcome the limitations of current ocular imaging methods in functional studies^21^. In simulation studies, for example, it has been shown that by increasing the vessel diameter and melanin concentration, the relative error of SO_2_ measurement in the scattering-based method increases. However, the SO_2_ measurement is insensitive to these parameters in PAM^18^. As these results suggest, PAM can potentially be a more accurate tool in quantifying retinal SO_2_. The disadvantage of PAM in ophthalmic imaging arises, however, from its need to be in contact with the ocular tissue^22^. This physical contact may increase the risk of infection and may cause patient discomfort. Additionally, it applies pressure to the eye and introduces barriers to oxygen diffusion that alters physiological and pathophysiological balance of ocular vasculature function^8^. Additionally, the use of an ultrasonic signal detection scheme poses challenges for combining the technology with other imaging modalities^22^.

Recently, our group at PhotoMedicine Labs demonstrated, for the first time, non-contact photoacoustic imaging of the ocular tissue in murine eye using photoacoustic remote sensing (PARS) microscopy^23^. Later, we combined the technology with OCT and applied it for in-vivo non-contact simultaneous imaging of the anterior segment in the mouse eye^8^. We showed the potential of the system for estimating oxygen saturation in the anterior segment of the mouse eye and in the vasculature around the iris. Building on the proven strength of the PARS technology for functional imaging, we further modified the system configuration to make it suitable for non-contact imaging of the rodent retina. To this end, the imaging optics of the system is modified, and customized eye models are employed to perform the initial calibration of the PARS imaging system. OCT, which is a well-established technique for retinal imaging, is used here to navigate the PARS imaging and demonstrate the optical capabilities of the imaging setup. To our knowledge, this is the first time a non-contact photoacoustic modality is used for in-vivo imaging of the retina. This work has the potential to advance the diagnosis and treatment of major retinal disorders.

## Method

### System architecture

**Figure 1:**
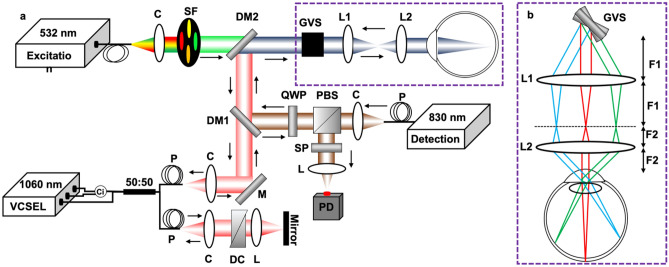
Retina imaging setup (**a**) Simplified schematic of the developed system. C: Collimator, Ci: Circulator, DC: Dispersion compensation, DM: Dichroic mirror, GVS: Galvo Scanner, L: Lens, P: Polarization controller, PBS: Polarized beam splitter, PD: Photodiode. QWP: Quarter wave plate, SF: Spectral Filter, SP: Short pass filter, VCSEL: Vertical Cavity Surface Emitting Laser. (**b**) Imaging optics arrangement of the telecentric pair for retina imaging. The distances between the elements are as F1 = 50 mm, F2 = 30 mm.

Figure 1a demonstrates the experimental setup of the system. A research-grade PARS-OCT system, designed and built by our group was modified and used in this study^8^. The developed system has two independent imaging modes. It can either performs as two standalone subsystems, or as a multimodal imaging unit to acquire simultaneous images. Here the PARS and OCT subsystems are used as two standalone imaging modalities, where the OCT is used to navigate the PARS imaging beams and verify the performance of the setup. Briefly, the output beam of a 532 nm ytterbium-doped laser (IPG Photonics) is coupled to a single mode optical fiber. At the output end of the fiber multiwavelength spectral peaks are generated through stimulated Raman scattering (SRS) with a pulse repetition rate (PRR) of 60 kHz^24^. The laser output was collimated into 2.1 mm diameter, then merged with the PARS probe beam centered at 830 nm (SLD830S-A20, Thorlabs) and the light of the swept source laser (center wavelength: 1060 nm; 100 nm spectral bandwidth corresponding to 7.3 µm axial resolution in biological tissue, 60 kHz swept rate, Thorlabs). The SNR of the system was measured ~ 100 dB at ~ 100 µm away from the zero-delay line with incident power of ~ 1.5 mW. A two-dimensional galvanometer scanned the combined light beams (GVS012/M, Thorlabs) and relayed them to the eye through a telescopic lens (50:30 ratio) system. In the PARS sub-system, pressure waves induced by light absorption were detected by the probe beam. The back-reflected light from the retina is directed towards the detection path. The photodiode outputs are connected to a high-speed digitizer (CSE1442, Gage Applied, Lockport, IL, USA) that performs analog to digital signal conversion. A comprehensive description of PARS detection mechanism, signal processing and image reconstruction techniques, can be found in previous reports of PARS imaging^25,26^. In the OCT system, the backscattered light from the retina first interfered with the reference arm beam, then detected by the built-in dual balanced photodetector. The OCT signal was digitized by a high-speed A/D card (ATS9351, Alazar Technologies Inc., Pointe-Claire, QC, Canada). The raw OCT data was transmitted to a host computer through a PCI‐Express interface. The system control was implemented in MATLAB platform to automatically control all the operations including system calibration, galvo‐scanning, system synchronization, real‐time imaging preview and data acquisition.

Due to the small size and thinner retina of rat and mouse eyes compared with human eyes, retinal imaging in these animal models is challenging. Two types of imaging interfaces have been widely used for small animal retinal imaging. One approach is to use a telecentric scanning configuration with a thin glass cover slip on the cornea to suppress corneal refraction^27^. The main advantage of this method is its relatively simple optical design which does not require detailed knowledge about the optics of the rodent eye. However, it increases intraocular pressure, and the achieved field of view is limited by the pupil diameter. Another approach is to use a scanning configuration with the scan pivot position located at the pupil. In this approach, a collimated beam is incident on the cornea, and it is focused on the retina by the optics of the murine eye. Therefore, a correct eye model must be used to achieve optimal performance. This pivoted scanning configuration has no risk of increasing intraocular pressure. Here, the field of view is limited by the pupil, and it is possible to move the field of view to the region of interest simply by adjusting the orientation of the murine eye with respect to the optical axis of the imaging interface^28^. In this study, the optimization of the telecentric configuration and initial calibration of the PARS setup was achieved through imaging human and rat eye models. A ratio of 50:30 was used for the telescopic pair and its pivot point was properly positioned to relay the galvanometer mirrors onto the entrance pupil of the eye. Figure 1b shows the pivoted scanning imaging interface used in our study. The incident collimated beams are ~ 1.2 mm diameter on the cornea. The lateral resolutions of both PARS and OCT were around ~ 6 µm in the retina with a slight difference caused by the shorter wavelength of PARS resulting in higher lateral resolution compared to the OCT system.

### Image reconstruction

All the PARS images shown in this manuscript were reconstructed using a maximum amplitude projection (MAP) of each A-scan for each pixel of the *en-face* image. The images were directly generated by plotting interpolated raw data using a Delaunay triangulation algorithm^29^. All images and signal processing steps were performed in the MATLAB environment.

For each OCT B-scan, 500 A‐lines were acquired with 2448 sampling points providing a depth ranging distance of ∼ 12 mm. To remove DC bias, reference spectrum was subtracted from the interference signal, then Fourier transform was performed to extract the depth‐resolved OCT signal. Images were generated from the raw OCT data and numerically dispersion compensated up to the 5th order with a custom MATLAB algorithm^30^. No additional image post-processing was used for the OCT images presented in this paper. The volumetric and en-face images were generated from the 3D data sets with ImageJ^31^.

### Animal preparation

All the experimental procedures were carried out in conformity with the laboratory animal protocol approved by the Research Ethics Committee at the University of Waterloo and adhered to the ARVO statement for use of animals in ophthalmic and vision research. All sections of this report adhere to the ARRIVE Guidelines for reporting animal research. Albino rats (Charles River, MA, USA) were imaged to demonstrate the in-vivo capabilities of the system. A custom-made animal holder was used to restrain the animal. The base of the animal holder was lined with a thermal pad to keep the animal body temperature between 36 and 38 °C. One drop 0.5% proparacaine hydrochloride (topical anesthetic; Alcaine, Alcon, Mississauga, ON, Canada) was applied to the eye, followed by one drop of 0.5% tropicamide (pupillary dilator; Alcon). Artificial tears were used frequently (~ every 2 min) to keep the cornea hydrated. Vital signs, such as respiration rates, heart rates and body temperature were monitored during the experiment.

## Results and discussion

### Phantom imaging

**Figure 2:**
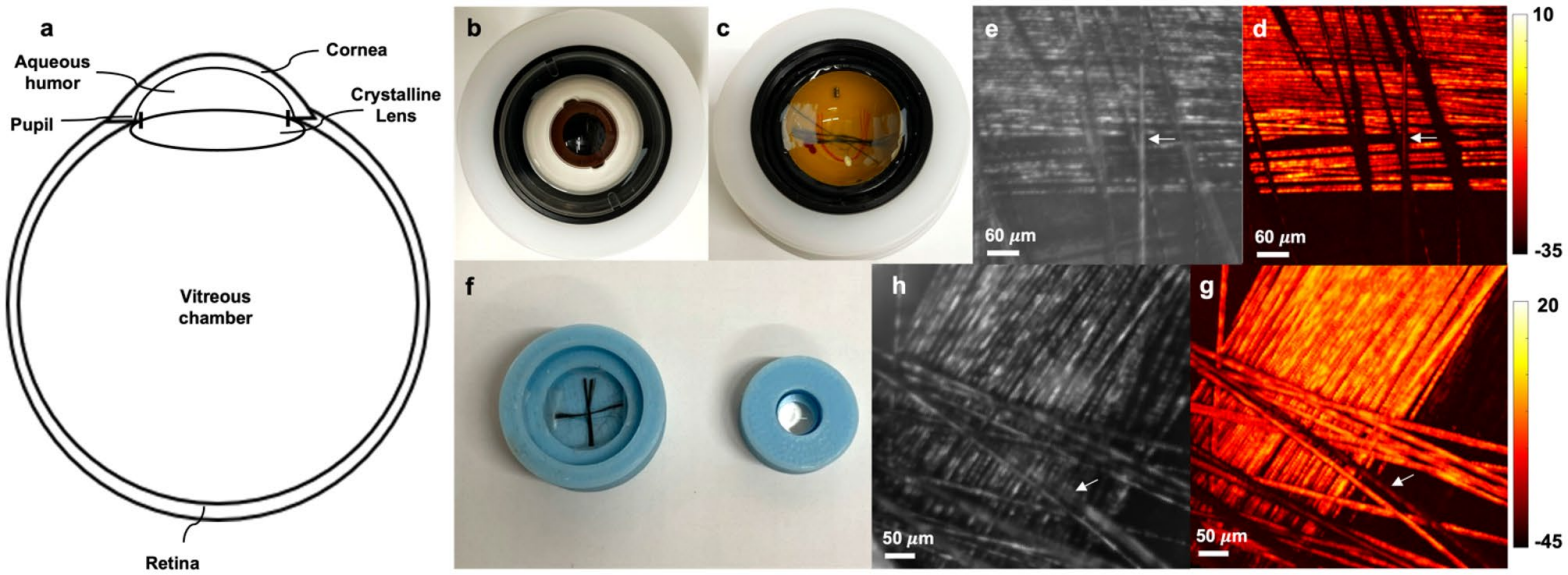
Imaging phantom eye models for human and rat. Simplified eye model consisting of the cornea, aqueous humor, pupil, crystalline lens, vitreous chamber, and the retina. (**a**) Human eye model (**b**). Strings of 7 μm carbon fibers are placed at the bottom of the eye model (**c**). CF image acquired with PARS scattering mechanism (**d**). CF image acquired using PARS absorption mechanism (**e**). Custom rat eye model consisting of a single achromatic lens and a 3D printed plastic chamber and strings of carbon fibers (**f**). PARS scattering and absorption contrast images, respectively (**g**, **h**). PARS excitation and detection beams are co-aligned so that the same CF are in focus in both images (white arrows).

Initial calibration of the PARS system was achieved through imaging eye phantoms. A simplified eye is shown in Fig. 2a, which is composed of the cornea, aqueous humor, pupil, crystalline lens, vitreous chamber, and the retina. First, we used a realistic human eye model to test the feasibility of PARS on the retina. The phantom is shown in Fig. 2b, and it was modified from a commercial product (OEM-7, Ocular Instruments, Bellevue, WA). The model consists of the cornea, crystalline lens, aqueous humor, vitreous humor, and artificial retina. Strings of 7 µm carbon fibers (CF) were placed at the bottom of the model where the artificial retina is located (Fig. 2c), and they were imaged using the PARS system. Figure 2e shows a representative image acquired using the PARS scattering mechanism described in our previous report^8^ and Fig. 2d shows the corresponding PARS absorption contrast image acquired from CF at the back of the human eye model. The PARS excitation and PARS detection are co-aligned, so that the same fibers are in focus in both absorption and scattering contrast images (white arrow).

Then a custom rat eye model was developed to help with the alignment of PARS excitation and detection beams for in-vivo trials. The eye model consisted of a single achromatic lens (63–817; Edmund Optics, Inc) with numerical aperture and curvature close to that of in the rat eye, and a custom 3D printed plastic chamber. Strings of 7 µm carbon fibers were located at a fix distance from the lens (Fig. 2f) corresponding to the focus of the lens and was approximately equal to the path length of a rat eye^32^. Representative images acquired using scattering and absorption contrast of PARS system are shown in Fig. 2g, h. Similar to the human eye model, the excitation and detection beams were co-aligned so that the same CF are in focus in both images (white arrows).

### SO_2_ accuracy

**Figure 3:**
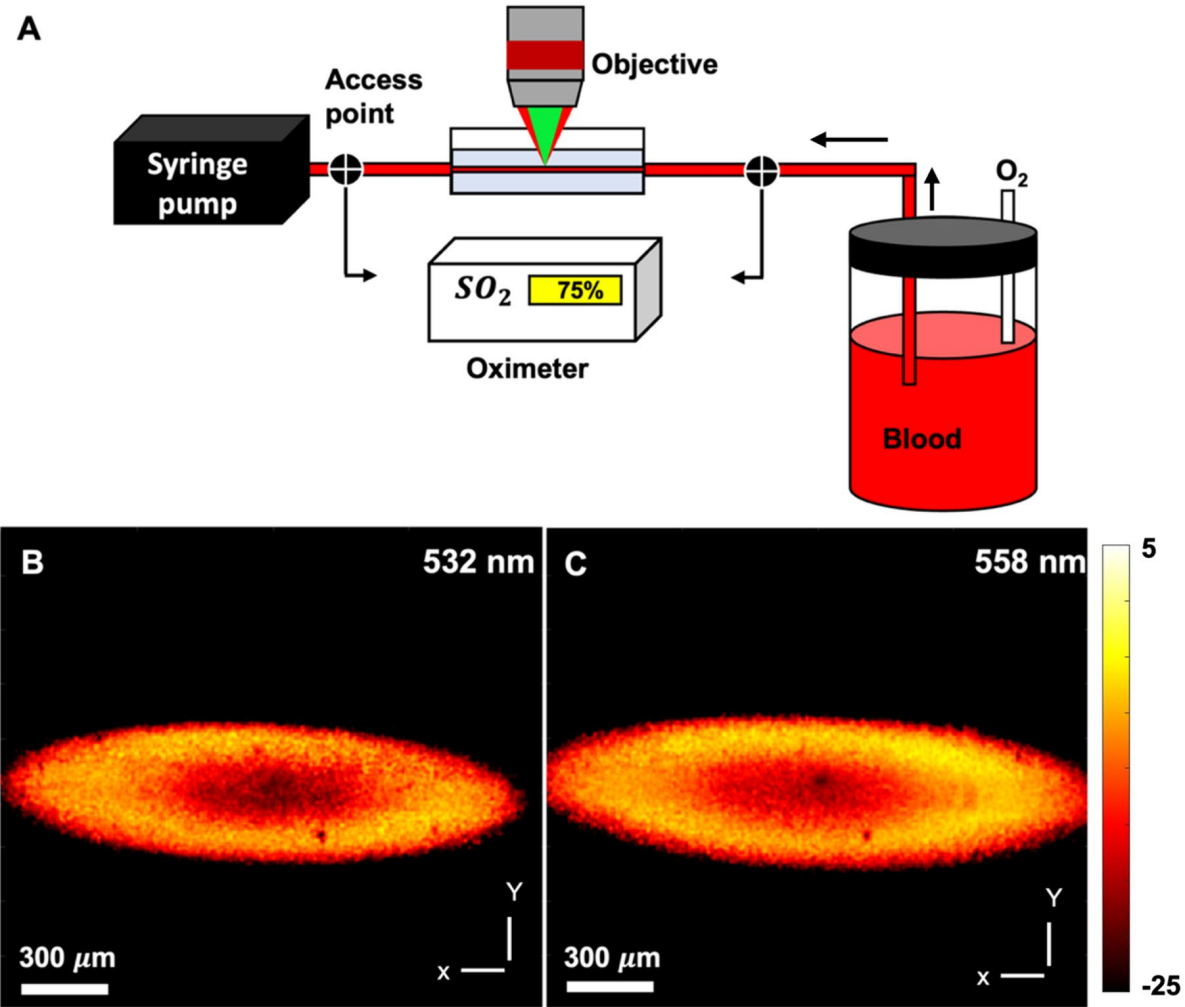
In-vitro bovine blood phantom experiment. Experimental setup of the experiment (**A**). Images of glass capillary with flowing blood acquired at 532 nm and 558 nm excitation wavelengths, respectively (**B**, **C**).

The accuracy of the multiwavelength PARS system in measuring oxygen saturation was investigated by performing In-vitro phantom experiments using freshly collected bovine blood with Sodium Citrated anticoagulant solution. Figure 3A shows the experimental setup. The setup included a blood reservoir, tubing, oxygen tank, oxygen meter access points and a syringe pump (NE-4000, New Era Pump Systems, Inc.). Different levels of oxygen were delivered to the blood reservoir. Blood samples were drawn before and after image acquisition for CO-oximetry measurements. A clinical grade CO-oximeter (Avoximeter 4000, Instrumentation Laboratory LTD, Richmond Hill, Canada) served as a reference device for measuring blood oxygen saturation.

Images of glass capillary with flowing blood are acquired at 532 nm and 558 nm excitation wavelength (Fig. 3B,C). To estimate the SO_2_, representative signal values were extracted from the two images. It is assumed that the SO_2_ is homogeneous within the capillary and the mean values are extracted. Compared to the per-pixel basis estimation, the mean value method offers higher accuracy by reducing the impact of random errors. Here, rather than averaging the signal intensity over the entire image, only a sub-region where the target is adequately in-focus is used. The PARS signal intensity within the selected region is averaged to arrive at a representative value associated with each excitation wavelength. The relative concentrations of oxy- and deoxyhemoglobin and the corresponding SO_2_ value is then calculated. Since the data acquisition time was fast (< 6 s), and the capillary tubing connections were airtight, the oximeter’s reads before and after each acquisition were almost identical (± 0.4%). The PARS measurements showed 40% and 60% oxygenation over 4 separate measurements, which were found to be within 6% accuracy with the oximeter results (measured as standard deviation of the acquired data). The value is in the range reported by other photoacoustic imaging studies^33,34^ and approaches the ISO-defined acceptable performance criterion for oximeters (< 4%)^35^. To further improve the accuracy several factors can be considered in future works. For example, nonlinear methods and fluence correction algorithms can be investigated to compensate for the absorption saturation effect and wavelength-dependent depth-coloring^36^. Recently, Liu et al. developed a photoacoustic microscopy system with three spectral wavelengths (532 nm, 545 nm, and 558 nm) and implemented a nonlinear algorithm to compute SO_2_^37^. They suggested that since blood has high absorption coefficients in the visible spectrum, it may cause absorption saturation and induce systematic errors. Therefore, nonlinear methods can compensate for the absorption saturation and improve the accuracy of the measurements. Mitcham et al. reported accuracy of 3–15% depending on use of fluence correction and different unmixing methods^38^. In another study by Laufer et al., SO_2_ accuracy of 2.5–4% was reported using a photoacoustic tomography technique^39^. They reported significant improvements in SO_2_ accuracy after fluence correction over a depth of 40 mm. However, the effect of spectral coloring was not significant for imaging depth < 5 mm, which is similar to the case reported in this study.

### Retina imaging

**Figure 4:**
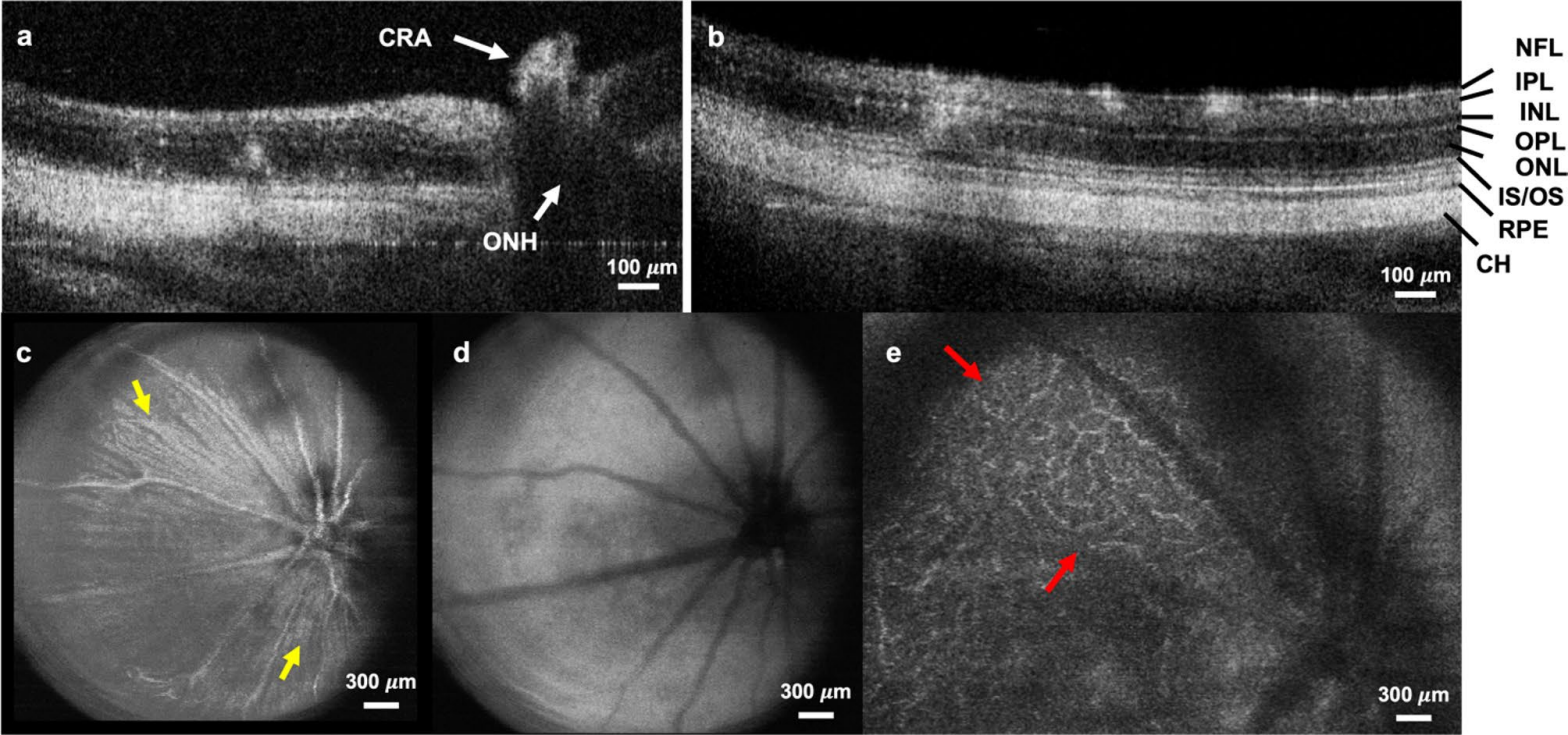
Volumetric and cross-sectional OCT images. Cross-sectional images acquired in-vivo from rat retina showing distinct layers of the retina. CH: choroid, CRA: central retinal artery, INL: inner nuclear layer, IPL: inner plexiform layer, IS/OS junction of inner segment and outer segment layer, NFL: nerve fiber layer, ONL: Outer nuclear layer, ONH: optic nerve head, OPL: outer plexiform layer, RPE retinal pigment epithelium layer. (**a**, **b**). OCT fundus images visualizing optic nerve head, large retinal vessels, optic nerve fiber bundle (yellow arrows), deeper retinal layer microvasculature (red arrows) (**c**–**e**).

First, the OCT system was applied to investigate the performance of the optical setup and navigate the PARS imaging beams. Cross-sectional and volumetric OCT images of the rat retina are demonstrated in Fig. 4. Each data set is acquired in ~ 10 s. Cross-sectional images enable visualization of major retinal layers. Retinal nerve fiber layer (NFL) and the retinal ganglion cell layer (GCL) form the innermost (top), highly reflective band. Beneath this are the inner plexiform (IPL), inner nuclear (INL), outer plexiform (OPL), and outer nuclear layer (ONL). In principle, those layers formed of nerve fibers, i.e., IPL and OPL show high-backscattering, whereas the nuclear layers have low-backscattering. The junction between the inner segment and outer segment of the photoreceptors could be visualized, as well as the highly reflective band which comprises the retinal pigment epithelium (RPE) (Fig. 4b). In the OCT images, the central retinal artery (CRA) remnants were also visible. (Fig. 4a). OCT fundus views are also generated by axially summing the merged OCT data set with a field-of-view of ~ 2.6 × 2.6 mm (Fig. 4c–e). From the OCT fundus image, as shown in Fig. 4C, specific anatomy of the rat retina is visualized, including the optic nerve head, some large retinal vessels, and optic nerve fiber bundles as indicated by the yellow arrows. Figure 4e shows the retinal microvasculature in the deeper retinal layer (Red arrows).

**Figure 5:**
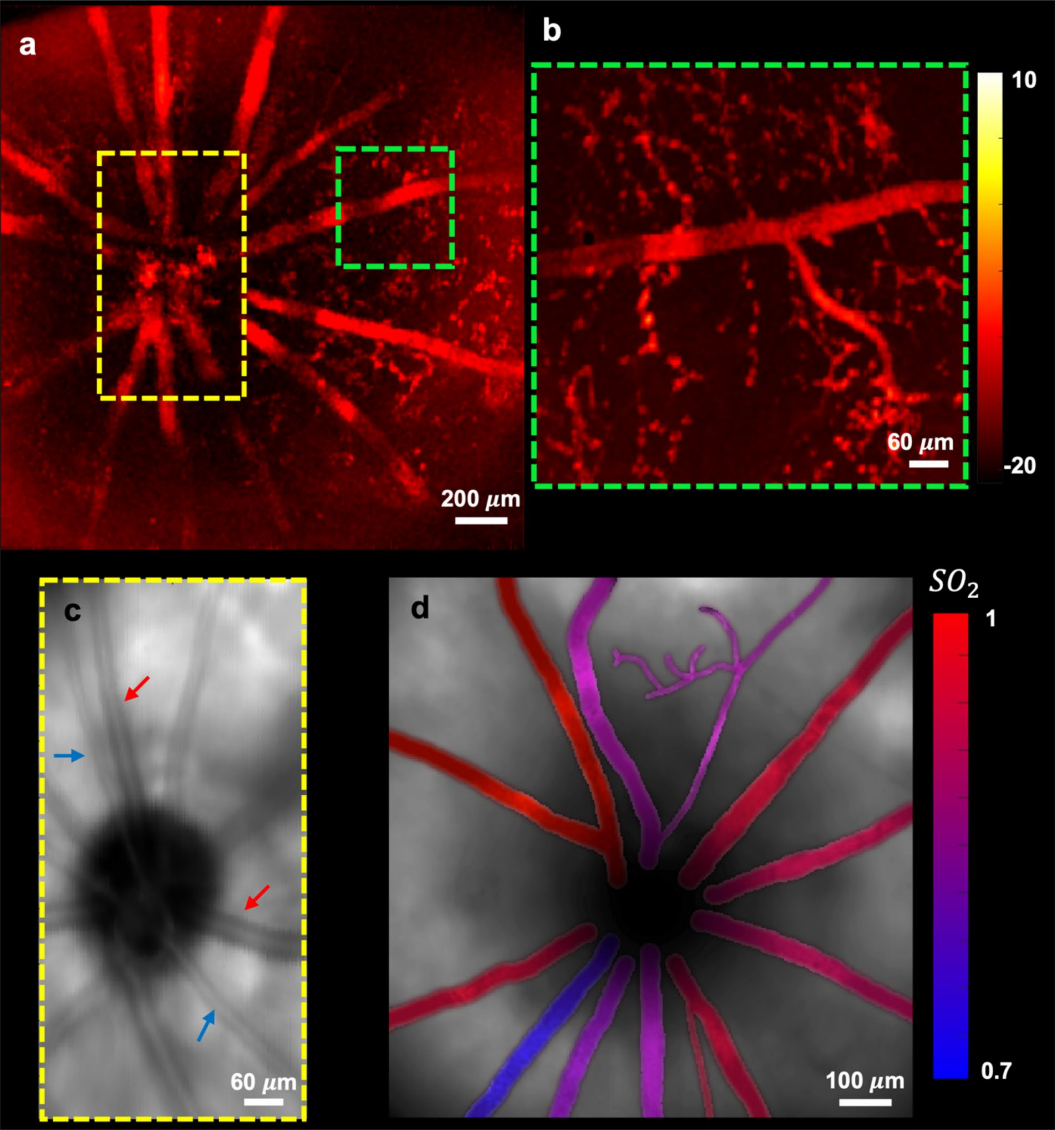
PARS retinal imaging. Fundus PARS image acquired from large vessels around ONH from a 2.6 × 2.6 mm area (**a**). zoomed-in section of one of the vessels acquired from a similar area with smaller vasculature (**b**). Fundus image acquired using scattering contrast of PARS system showing arteries (red arrows) and veins (blue arrows) (**c**). Oxygen saturation map in the retina obtained using multiwavelength PARS imaging (**d**).

After verifying the optical performance of the setup, the PARS imaging system was used to image rat retina. The axial resolution of the PARS system (~ 40 µm), enables separating the signals of retinal blood vasculature from the RPE melanin given the retina thickness of ~ 200 µm^17^. As a result, PARS signals generated from major vessels can be considered mainly from hemoglobin. Figure 5a demonstrates fundus PARS image acquired from large vessels around ONH from a 2.6 × 2.6 mm area. The SNR of the image was measured as 26 ± 3 dB. Smaller vessels (~ 20 μm) are also visible in the image; however, they are slightly distorted by motion artifacts. The boxes in this figure do not highlight the exact structure and are used only to show the approximate region imaged. Figure 5b shows a zoomed-in section of one of the vessels acquired from a similar area where the smaller vasculature is more visible. In the previous report of the system^8^, the PARS scattering contrast provided through the PARS detection beam was introduced. In retinal imaging applications, this capability allows the PARS microscope to be used the same way as fundus photography to image the interior surface of the eye. Figure 5c, shows a representative image acquired from the ONH using scattering contrast of the PARS system. Since the 830 nm beam used in the proposed architecture has different absorption coefficients for oxygenated and de-oxygenated hemoglobin (higher absorption for oxygenated hemoglobin), arteries appear dark (red arrows), and veins are light (blue arrows) in the image. Therefore, the scattering contrast of the PARS microscope can be used similarly to retinal oximeters to measure oxygen saturation in the eye^40^.

Relying on the capability of the PARS system to provide functional information, the amplitude of multiwavelength PARS signals were employed to estimate SO_2_ in every vessel (Fig. 5d). The calculation is done based on the molecular extinction coefficients of HbO_2_ and Hb at 532 nm and 558 nm optical wavelengths. The rodent’s venous SO_2_ measured with PARS was around 70% which agrees with previous reports^41^. Despite the good match between measured SO_2_ by PARS and the preset SO_2_ for blood experiments, the in-vivo rodent experiments conditions are different from those in In-vitro experiments. For example, there are eye movements during imaging, notable chromatic aberration from the eyeball, etc. Whether such differences will affect the SO_2_ quantification or not requires future validation^42,43^. Additionally, it is reported that visual stimulus might increase the retinal vessel diameter and blood flow, as well as affect the retinal SO_2_ due to retinal neurovascular coupling^44^. These factors may affect the accuracy of the SO_2_ measurements, and they need further investigation in the future. Currently, retinal oximeter devices provide only an estimation of retinal oxygen saturation. In the absence of ground truth, results reported by these techniques fail to fully address the accuracy requirements of the measurements. Additional research is necessary to demonstrate the reliability of the proposed method at all saturation levels and across a diverse patient population. To our knowledge, this is the first report of optical absorption based retinal SO_2_ demonstrated in the fundus view. It is important to note that PARS can provide two independent absorption contrast effect. First, is the absorption contrast provided by the excitation laser. In this case, an isosbestic detection laser wavelength can be used to only visualize the excitation effect. Finally, PARS detection laser can also act as the secondary absorption contrast (Fig. 5c.). The combination of these two effects, which is unique to PARS microscopy, can lead to more accurate measurements compared to current methods. This will be investigated further in the future.

### Ocular light safety

Using the ANSI standards, the ocular light safety for the developed system can be calculated^45,46^. First, the safety of PARS excitation laser is tested using the three ANSI rules: single pulse limit, average power limit, and multiple pulse limit. The pulse duration for the IPG laser used in this study is $t_{1} = 1.5 \,{\text{ns}}$. In the imaging setup, assuming that the collimated laser beam has a maximum diameter of 20 × 2.6 µm on the retina after being focused by the eye, the angular subtense of the source:

$$ \alpha = \frac{20 \,\upmu {\text {m}}}{{17 \,{\text{mm}}}} = 1.2 \,{\text{mrad}} < \alpha_{{{\text{min}}}} = 1.5 \,{\text{mrad}} $$

where 17 mm is the focal length of human eye. Therefore, the light source can be considered as a point source.

- *Rule 1* Since the pulse duration of the laser is $1.5\, {\text{ns}}$ only the thermal effect needs to be considered and the maximum permissible exposure (MPE) will be:
  $$ MPE_{SP} = 5 \times 10^{ - 7} \, {\text{J}}/{\text{cm}}^{2} $$
- *Rule 2* Since the exposure time ($\sim 10 s$) is longer than the $0.7 s$ and the wavelength is between 400 and 600 nm, dual limits due to both thermal and photochemical effects apply here. For the photochemical effect:
  $$ MPE_{ph} = 4.36 \times 10^{ - 2} \,{\text{J}}/{\text{cm}}^{2} $$
  For the thermal effect:
  $$ MPE_{th} = 1.01 \times 10^{ - 2} \,{\text{J}}/{\text{cm}}^{2} $$
- *Rule 3* tests whether an exposure by a long pulse of duration $nt_{1}$ is safe. Within a laser spot of 20 µm, there are at most two overlapping laser pulses (n = 2).
  $$ MPE_{rp} = n^{ - 0.25} \times MPE_{sp} = 0.84 \times 5 \times 10^{ - 7} = 4.2 \times 10^{ - 7} \,{\text{J}}/{\text{cm}}^{2} $$

Rule 3 is the most conservative of the three. Considering a pupil diameter of $D = 0.7\, {\text{cm}}$ MPE for a single pulse would be equal to $MPE_{rP} \times \left( \frac{D}{2} \right)^{2} \times \pi \approx 160 \,{\text{nJ}}.$ The acquired value is in correspondence to the values reported by other groups^47,48^. In this manuscript, the energy of a single pulse is $< 150\, {\text{nJ}},$ which is below the allowed pulse exposure limit.

For $\lambda$ = 380–1400 nm, a spectrally flat limit was recently introduced that recommends MP corneal irradiances of 25t^−0.75^ W/cm^2^ for $t < 10 s, (t = 10, MP = 4.4$ W/cm^2^), and 4.0 W/cm^2^ for $t > 10s$ ^49^. Based on the proposed limit the OCT light power on the cornea ( $\sim 1.5 \,{\text{mW}}$) and the PARS detection power ($\sim 2\,{\text{to}}\,3 \,{\text{mW}})$ are well within the ANSI limits.

There are several aspects of the proposed system that can be further refined for future studies.

First, the current PARS-OCT system was developed using visible excitation wavelength for targeting oxygenated and de-oxygenated hemoglobin in the blood. However, the laser safety threshold for ocular imaging is stricter in the visible than in the NIR spectral range. Additionally, the retina is sensitive to the visible light, which might cause neurovascular coupling and affect the oxygenation as well as pose challenges to eye fixation during imaging. Therefore, to overcome these issues in future studies, the NIR spectral range can be examined as the excitation wavelength.

Second, the presented results in this study suffered from common problems associated with imaging of anesthetized animals including tear film breakup, drying of the corneas, and development of cold cataracts. These effects can be overcome by the use of a contact lens and transparent gel to maintain clarity and moisture in the cornea, and by maintaining and monitoring body temperature during imaging^50^. Most eye movements, including involuntary drift and saccades, are not present in rats under anesthesia. However, due to the eye motion caused by animal breathing and consequent head movements, there are still motion artifacts in the images. These motion artifacts can be prevented by employing a proper stereotactic animal holder. They can also be corrected in the post image processing stage.

Third, the oxygen saturation measurement accuracy can be improved by employing a more stable tunable light source, using three or more excitation wavelengths, and applying nonlinear processing techniques. Challenges remain in properly accounting for pigmentation, scatter, and spectral properties of the anterior optics that may alter or bias spectral measurements and degrade the integrity of the oximetric signal^51^. To test the full capability of the proposed system and how its accuracy is affected by these parameters requires further investigations.

In future, the multimodal capability of the system can be employed to enable simultaneous PARS-OCT imaging that could significantly increase the insight into retinal pathology. For example, the scanning pattern of the OCT system can be modified to enable Doppler OCT and OCT angiography and provide blood flow measurement and vasculature map, respectively. This information can be further combined with the non-contact oxygen saturation measurements of PARS to measure metabolic rate of oxygen consumption in the ocular environment in both small and large vessels. The system can be further applied for evaluating the effects of visually evoked stimulus and how they could change the retinal activity. It can also be used in longitudinal studies on larger animal models like rabbit and monkeys with size of eyeballs closer to that of humans. Additionally, the current design of PARS microscopy reads out phase-insensitive intensity reflectivity, and the detected signals do not produce depth-resolved information^25^. Therefore, OCT can add the depth-resolved scattering information to the chromophore selected absorption information of PARS and provide a comprehensive map of the tissue structure and functional properties. Also, the feasibility of using a single light source for PARS and OCT imaging can be explored in future studies. This would reduce the complexity and costs of the system and lower the optical power entering the eye. Additionally, it will be easier to generate synchronized and co-registered PARS and OCT images.

## Conclusion

In conclusion, for the first time, a non-contact photoacoustic imaging technique was applied for in-vivo measuring of retinal SO_2_. It was also the first time in-vivo imaging of retina was demonstrated using a non-contact photoacoustic imaging modality. The proposed method can be a measure step toward non-invasive measurement of metabolic rate of oxygen consumption in retina, and it can further improve the diagnosis and treatment of important eye diseases.
