# Author Correction: In-vivo functional and structural retinal imaging using multiwavelength photoacoustic remote sensing microscopy

**DOI:** 10.1038/s41598-022-09412-5

**Published:** 2022-03-29

**Authors:** Zohreh Hosseinaee, Nicholas Pellegrino, Nima Abbasi, Tara Amiri, James A. Tummon Simmons, Paul Fieguth, Parsin Haji Reza

**Affiliations:** 1 PhotoMedicine Labs, Department of System Design Engineering, University of Waterloo, 200 University Ave W, Waterloo, ON N2L 3G1 Canada; 2 Department of System Design Engineering, University of Waterloo, 200 University Ave W, Waterloo, ON N2L 3G1 Canada

Correction to: *Scientific Reports* https://doi.org/10.1038/s41598-022-08508-2, published online 16 March 2022

The original version of this Article contained an error in the Methods section, under the subheading ‘Animal preparation’,

“Albino rats (NU/NU, Charles River, MA, USA) were imaged to demonstrate the in-vivo capabilities of the system.”

now reads:

“Albino rats (Charles River, MA, USA) were imaged to demonstrate the in-vivo capabilities of the system.”

The original Article has been corrected.

